# Active Thermoelectric Vacuum Sensor Based on Frequency Modulation

**DOI:** 10.3390/mi11010015

**Published:** 2019-12-21

**Authors:** Shu-Jung Chen, Yung-Chuan Wu

**Affiliations:** Department of Mechatronics Engineering, National Changhua University of Education, Changhua City 50074, Taiwan; yungchuan@gmail.com

**Keywords:** thermoelectric-type vacuum sensor, vacuum detection, frequency modulation

## Abstract

This paper introduces a thermoelectric-type sensor with a built-in heater as an alternative approach to the measurement of vacuum pressure based on frequency modulation. The proposed sensor is fabricated using the TSMC (Taiwan Semiconductor Manufacturing Company, Hsinchu, Taiwan) 0.35 μm complementary metal-oxide-semiconductor-microelectro-mechanical systems (CMOS–MEMS) process with thermocouples positioned central-symmetrically. The proposed frequency modulation technique involves locking the sensor output signal at a given frequency using a phase-lock-loop (PLL) amplifier to increase the signal-to-noise ratio (SNR) and thereby enhance the sensitivity of vacuum measurements. An improved first harmonic signal detection based on asymmetrical applied heating gives a precise measurement. Following calibration, the output voltage is in good agreement with the calibration values, resulting in an error of 0.25% under pressures between 0.1–10 Torr.

## 1. Introduction

Advances in micromachining and the miniaturization of electronic circuits has necessitated the development of ever smaller vacuum sensors [1,2]. Microelectronic devices and microelectro-mechanical systems (MEMS) are now easily integrated on the same chip to accelerate data processing, reduce fabrication costs, and improve energy efficiency. The current trend in sensing technology focuses on SOC (system on a chip) schemes, due to advances in the fabrication of semiconductors and the ease with which such algorithms can be developed.

A complementary metal-oxide-semiconductor (CMOS)–MEMS has proven particularly effective in the development of SOC technology, due to the maturity of the processes and its compatibility with standard CMOS technology [3,4,5]. Thermal micro-sensors for the measurement of vacuum pressure have proven highly effective in terms of pressure range, accuracy, and reliability [6,7,8,9,10,11], and this in turn has prompted efforts to develop these devices using CMOS–MEMS technology to facilitate installation [12,13].

Sensors based on thermoelectric effects have attracted considerable attention in recent decades [14,15,16,17]. Thermopiles, based on the Seebeck effect, have been used for the monitoring of gas flow, vacuum pressure, and even as accelerometers [8,9,10,12,15]. These devices can be produced using batch-processing and researches have presented a broad range of commercial applications [10,18]. Most importantly, thermopile-based vacuum sensors are fully compatible with CMOS processes, thereby enabling high yield production with minimal post-MEMS processing [6,7,10]. A number of high-sensitivity vacuum sensors also have been developed through the etching of silicon substrates and the application of thin film processes [19].

During the current study, we develop an active thermopile using TSMC (Taiwan Semiconductor Manufacturing Company, Hsinchu, Taiwan) 0.35 μm 2P4M (2 poly, 4 metal layers) standard CMOS semiconductor processing for vacuum measurement. Unlike conventional thermoelectric components, we built a heater into the sensing area of the CMOS thermopile to provide a heat source. During operation, the exchange of gaseous molecules between the heater and heat sink determines the working temperature on the hot junction of thermoelectric elements. Along these lines, the pressure of the gas within the chamber influences the difference in temperature between the hot and cold junctions in the thermoelectric element, which in turn leads to a small variation in voltage. The thermopile comprises multiple thermoelectric elements (in series), which generate an output signal by which to measure vacuum pressure. Under frequency modulation, we propose an improved first harmonic signal detection and mathematical model of gas thermal conductance based on asymmetrical applied heating to acquire the signal precisely from the thermopile. The weak signal from the thermopile is first amplified using an inverting amplifier circuit and then modulated using a phase-lock-loop (PLL) amplifier. Our developed CMOS–MEMS vacuum sensor is presented in the following section. The working principle of a thermoelectric-type sensor for vacuum MEMS and its key issue for vacuum measurement are described. The vacuum measurement systems based on frequency modulation are built and measured results are analyzed and discussed.

## 2. Working Principle and Design of Proposed Metal-N-Poly Thermoelectric-Type Sensor

The performance of the micro-sensors in measuring vacuum pressure depends on various factors. It is important to enhance the signal-to-noise ratio (SNR) of vacuum measurements by developing the frequency modulation technique and to minimize the heat dissipation via conduction from the sensing area to the main substrate is the key issue for the performance of the sensor.

### 2.1. Sensing Scheme for Thermoelectric-Type Sensor Based on Frequency Modulation

**Figure 1:**
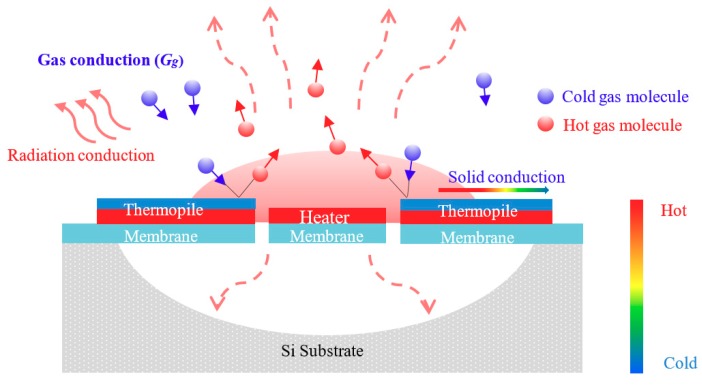
Sensing scheme of proposed thermoelectric-type sensor.

Heat dissipated from the heater membrane to the silicon substrate via thermal conduction through the membrane structure as well as flow convection through collisions of the surrounding gas molecules. Radiation will contribute to the total thermal conductance only in the case of a high temperature and is omitted here [20]. Figure 1 presents the sensing scheme of the proposed thermoelectric-type sensor under the three forms of heat transfer.

Seen in a steady-state condition, the temperature of the thermopile sensor increases under the effects of electrical heating power (*P_e_*) from the poly-silicon heater subjected to an applied current (*I*), the value of which can be calculated as follows:

(1) $$P_{e}=I^{2}R=\frac{V^{2}}{R}$$

where *V* and *R* respectively refer to the applied voltage and resistance of the thermopile sensor. Looking at a conventional lumped model, the thermal behavior of a sensor is dominated by the exchange of heat between the sensor and the surrounding environment. Thus, the behavior of the sensor is governed by the following equation:

(2) $$H\frac{dT}{dt}+G(T-T_{a})=P_{e}$$

where *H* and *G* respectively refer to the heat capacity and total thermal conductance of the sensor, whereas *T* indicates the working temperature and *T_a_* indicates the ambient temperature. The bias voltage *V*(*t*) of a harmonic waveform with an offset which is asymmetrically applied to the poly-silicon heater is as follows:

(3) $$V(t)=V_{b}\sin(2\pi ft)+V_{\mathrm{off}}$$

where *V_b_* refers to the amplitude of the sinusoidal function of heating under a constant offset voltage (*V*_off_), whereas *f* and *t* are the modulation frequency and time of heating. Thus, with the addition of heating power, Equation (2) is reformulated as follows:

(4) $$\begin{array}{l} H\frac{dT}{dt}+G(T-T_{a})=\frac{V_{b}^{2}}{R}{\left(\sin(2\pi ft)+\frac{V_{\mathrm{off}}}{V_{b}}\right)}^{2}\\ =\frac{V_{b}^{2}}{R}\left(\frac{-1}{2}\cos(4\pi ft)+\frac{2V_{\mathrm{off}}}{V_{b}}\sin(2\pi ft)+{\left(\frac{V_{\mathrm{off}}}{V_{b}}\right)}^{2}+\frac{1}{2}\right) \end{array}$$

Gas conductance *G_g_* under pressure *P* dominates the response of the proposed thermoelectric vacuum sensor described as follows:

(5) $$G_{g}(P)=\frac{\varphi }{2-\varphi }A_{s}G_{a}P(\frac{P_{t}}{P+P_{t}})$$

(6) $$G_{a}=\Lambda_{o}{\left(\frac{273.15}{T_{a}}\right)}^{1/2}$$

where *ϕ* is the accommodation coefficient of gas; *A_s_* is the area of floating membrane; *G_a_* and *Λ_o_* are the free molecular thermal conductivities at *T_a_* and at 273 K, respectively. Note that this value is determined by the type of gas and the specific properties of the materials used in the sensor, as well as the configuration in details. *P_t_* refers to the transition pressure associated with the transfer of heat [20], which is the pressure used to characterize the mechanisms underlying the thermal conductivity of the gas in the chamber.

The total thermal conductance of the thermopile sensor and its relation to pressure are described as follows:

(7) $$G(P)=G_{s}+G_{g}(P)+G_{r}$$

The corresponding solution for the heat equation includes three terms, which can be expressed as follows:

(8) $$T(t)=T_{o}+T_{1}\sin(2\pi ft+\phi_{1})+T_{2}\sin(4\pi ft+\phi_{2})$$

Under frequency modulation, the measured signal is locked at frequency *f*, such that only the amplitude *T*_1_ is read from the PLL.

(9) $$T_{1}=T_{1}(V_{b},P,f)=\frac{\frac{2}{G(P)}\left(\frac{V_{b}^{2}}{R}\right)\left(\frac{V_{\mathrm{off}}}{V_{b}}\right)}{\sqrt{1+{\left(2\pi f\tau \right)}^{2}}}$$

where *τ* (= *H*/*G*) is the thermal time constant of the sensor.

The original signal *V*_th_ from the thermoelectric sensor is derived from the difference in temperature Δ*T* = *T*(*t*) − *T_a_* and then amplified using an expression describing the transient response of the output signal *V*_out_ (*t*), as follows:

(10) $$V_{\mathrm{out}}(t)=a\cdot V_{\mathrm{th}}(t)+b=a\cdot \Delta S\cdot \Delta T(t)+b$$

where coefficients *a* and *b* are the gain of the amplification and offset of the sensing circuit; Δ*S* is the difference between the Seebeck coefficients of the two types of material used in the fabrication of the thermopile structure.

The output signal *V*_out_ is rewritten with three terms (a constant, the first harmonic term, and the second harmonic terms), as follows:

(11) $$V_{\mathrm{out}}(t)=V_{0}+V_{1}\sin(2\pi ft+\phi_{1})+V_{2}\sin(4\pi ft+\phi_{2})$$

Using the signal from the PLL locked at the first harmonic frequency *f*, the amplitude of the first harmonic term, *V*_1_ can be expressed using the dependent factors as follows:

(12) $$V_{1}=V_{1}(V_{b},P,f)=a\cdot \Delta S\cdot T_{1}(V_{b},P,f)$$

Overall, substituting Equations (7) and (9) into Equation (12) yields the following:

(13) $$V_{1}(V_{b},P,f)=\frac{\frac{2a\Delta S}{G_{s}+G_{g}(P)+G_{r}}\left(\frac{V_{b}^{2}}{R}\right)\left(\frac{V_{off}}{V_{b}}\right)}{\sqrt{1+{\left(2\pi f\tau \right)}^{2}}}$$

Note that *V*_1_ would be null without *V*_off_ in Equation (13). The application of offset voltage *V*_off_ becomes a key issue to facilitate the acquisition of the output voltage from PLL at the first harmonic frequency *f*. An asymmetrical bias input voltage of a sinusoidal wave is formed when *V*_off_ is set, thereby enabling the output signal of the thermopile to be measured precisely. This proposed approach highlights an efficient measurement when the measured signal is locked at frequency *f* under frequency modulation. To acquire *V*_1_, *V*_off_ in Equation (3) has to be set to form an asymmetrical bias input voltage for the active heater in the experiment.

An increase in the bias voltage applied to the heater *V*(*t*) should lead to a linear increase in *V*_1_. This means that the derivative of *V*_1_ with respect to *V_b_* should be independent of *V_b_*. This can be rewritten in a simpler form as follows:

(14) $$\frac{\partial V_{1}(V_{b},P,f)}{\partial V_{b}}=\frac{V_{1}}{V_{b}}=\Delta (P,f)$$

Regarding fixed conditions of *P*, *f*, the ratio of *V*_1_ to *V_b_* will keep a constant, which is irrelevant to *V_b_*.

### 2.2. Design and Fabrication of Thermoelectric-Type Sensor

**Figure 2:**
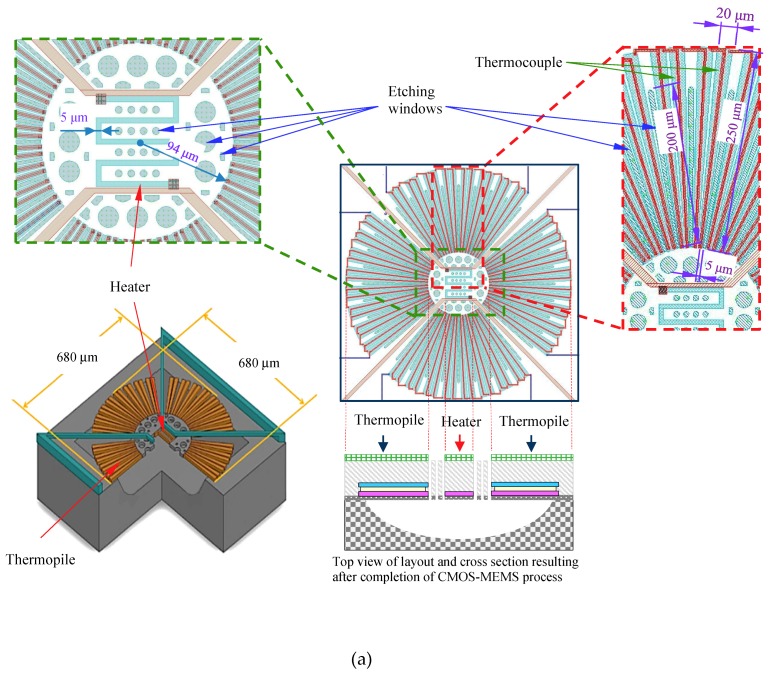

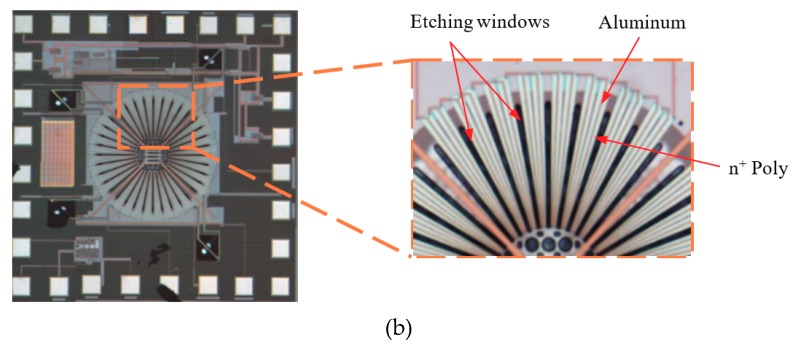
(**a**) Schematic drawing of the thermopile structure; (**b**) Photography of chip after fabrication.

To obtain a large active area for vacuum sensing, an active area of the thermopile with 680 × 680 μm^2^ was designed and patterned. Shown in Figure 2, the structure of the thermopile comprised 64 pairs of thermocouples (in four interconnected blocks) arranged in a central-symmetrical configuration surrounding a sensing membrane. Note that the thermocouples were fabricated using standard CMOS materials, metal (aluminum) and N-polysilicon (n^+^ Poly), connected in series. The patterning of the N-polysilicon was designed carefully and evaluated to minimize resistance, Johnson’s noise, and thermal conductance to maximize the signal-to-noise (SNR). Long, narrow, nearly triangular thermocouple strips were arranged as a star-shaped array on the circular membrane with the narrow end (width = 5 μm) facing the center and flaring outward toward the perimeter (maximum width = 20 μm). The length of the thermocouple (distance extending outward) was approximately 250 μm. The width of metal was 3 μm. An N-polysilicon heater (5 μm × 460 μm) with a heating radius of 94 μm was placed in the center of a thermopile structure. 

**Figure 3:**
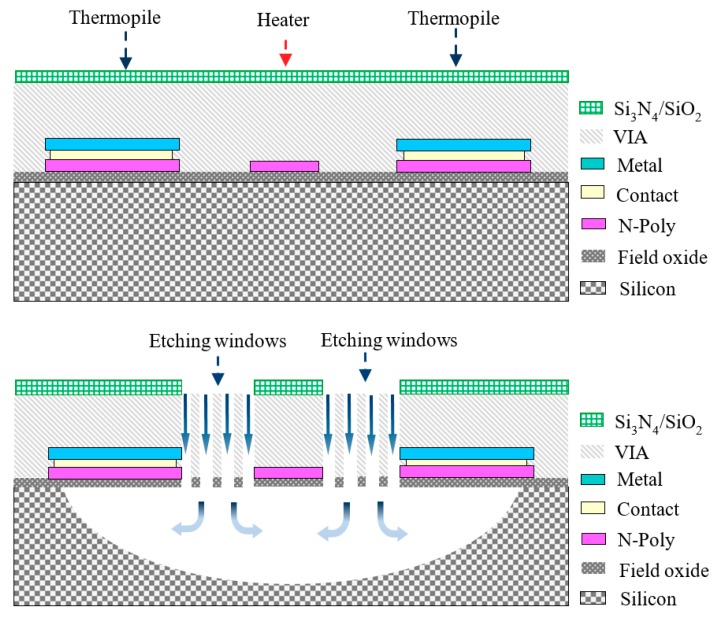
Fabrication steps of proposed sensor using a complementary metal-oxide-semiconductor (CMOS)-compatible process.

The performance of the thermal-type pressure sensor is promoted by reducing the heat conduction and increasing the contribution of the gas convection. This was achieved by a number of etching windows (length = 200 μm; average width = 4.8 μm) in the active area to allow the etchant to flow through the etching windows efficiently, which was helpful in realization of a freely suspended microstructure. After the CMOS processes, a series of post-processes, including an isotropic RIE (Reactive ion etching) and wet etching processes, also were used to remove the silicon beneath the sensing structures. Figure 3 illustrates the steps used to fabricate the thermoelectric-type vacuum sensor with active heater.

The total resistance of the entire structure (all four blocks) was measured at 14.8 kΩ, which translates to a resistance of 3.7 kΩ for each block. The design parameters of the thermoelectric vacuum sensor are summarized in Table 1.

## 3. Vacuum Measurement

### 3.1. Experiment Setup

**Figure 4:**
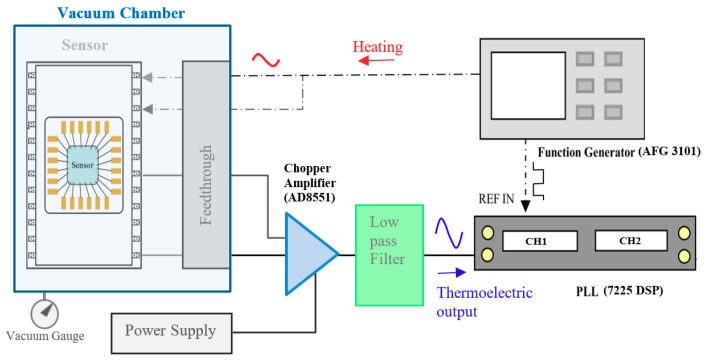
Setup of vacuum chamber and signal processing architecture.

Shown in Figure 4, the experiment setup included a vacuum chamber, a vacuum sensor, a function generator (AFG 3101, Tektronix, Beaverton, Oregon, USA), a signal processing circuit with a chopper amplifier (AD8551, Analog Devices, Norfolk, Massachusetts, USA) and low-pass filter, and a PLL amplifier (7225 DSP Lock-in Amplifier, Signal Recovery, Oak Ridge, Tennessee, USA). A conventional vacuum gauge was used to obtain reference values in calibration. The sensor installed within the vacuum chamber was connected to an amplification circuit and function generator using a four-wire feedthrough on the chamber wall.

### 3.2. Signal Acquisition from Thermoelectric-Type Sensor

The driving current for heating is delivered directly to the heater via a sinusoidal wave. A reference signal is also sent to the REF (reference) terminal of the PLL amplifier as a tracking signal. The signal processing circuit includes a chopper amplifier (AD8551) and PLL, as shown in Figure 4. The thermoelectric output signal is a weak sinusoidal wave with phase delay for the detection of temperature changes in the sensing area. After amplification and low-pass filtering, the signal is delivered to one of the input channels of the PLL amplifier. We set a time constant of 20 seconds to ensure stability in the amplitude and phase of the PLL for frequency measurement.

## 4. Results and Analysis of Vacuum Measurement

### 4.1. Frequency Response

**Figure 5:**
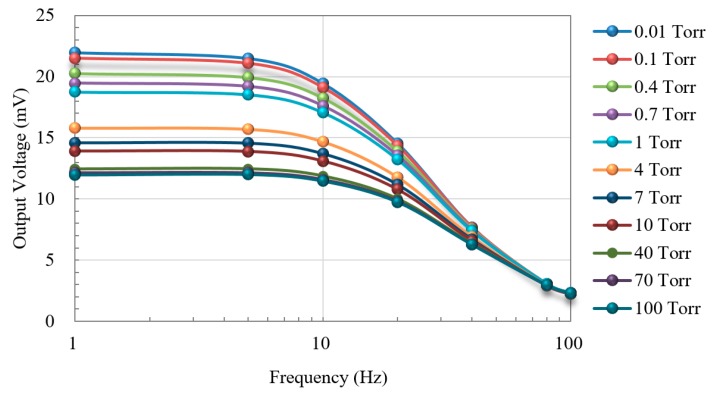
Output voltage versus frequency of input bias voltage applied to the heater.

During pressure measurements, the vacuum chamber was maintained between 0.01 and 100 Torr. It took about 30 minutes for the pressure in the vacuum chamber to stabilize. The heater in the proposed thermoelectric sensor was operated under a modulated first harmonic frequency of 1–100 Hz. When setting up the function generator, the amplitude of the sinusoidal bias voltage for the heater was fixed at 2 V, whereas the offset voltage was set as 0.26 V to give an asymmetrical bias voltage for heating the active heater. The output signal *V*out was then measured by the PLL amplifier to determine the frequency response. Increases in heating frequency led to a corresponding decrease in the signal from the PLL amplifier, as shown in Figure 5. One can see this presents the same characteristic curve associated with conventional thermal sensors.

Shown in Figure 5, when the input frequency exceeded 10 Hz, the output voltage dropped sharply, and when the frequency exceeded 60 Hz, the output voltage for different vacuum conditions was nearly the same without obvious difference. It reveals that the vacuum measurement is suggested to operate with modulation of heating at a low frequency, especially below 10 Hz for our proposed thermoelectric sensor.

It also is interesting to highlight the features and compare the modulation method of the first harmonic frequency with direct current (DC) measurement. While the modulation frequency reduces, the output voltage will approach the signal level of DC measurement, as shown by the flat band of the curves in the Figure. However, it is a practical design consideration of frequency bandwidth since the background noise and low frequency temperature drift with DC measurement will damage the performance.

### 4.2. Modeling Under-Frequency Modulation

**Figure 6:**
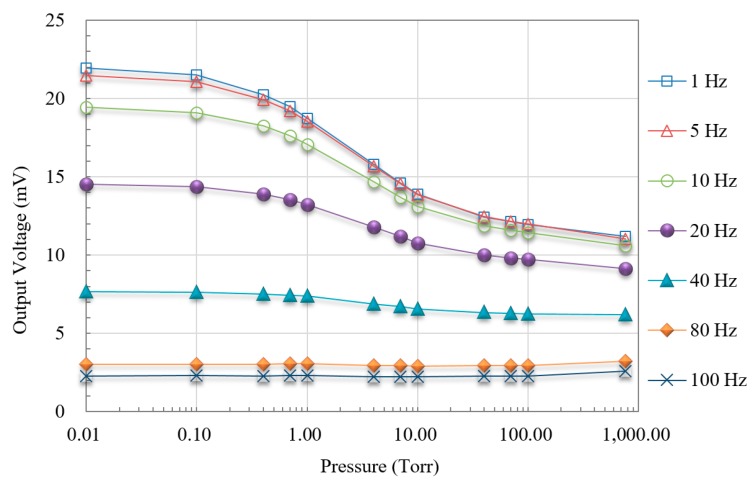
Output voltage versus pressure under different modulation frequencies.

Reliable pressure measurements can only be obtained by analyzing the signal as a function of pressure under various vacuum conditions. Figure 6 presents the voltage output by the PLL amplifier as a function of modulation frequency under various pressures.

When the heating first harmonic modulation frequency was less than 10 Hz (particularly at 1 Hz), the response to change in vacuum pressure from 0.01 to 760 Torr appeared as an S curve obviously, as shown in Figure 6. When the frequency exceeded 60 Hz, the slope of the signal curve was negligible. This is further evidence that the heating frequency should be maintained at less than 10 Hz.

**Figure 7:**
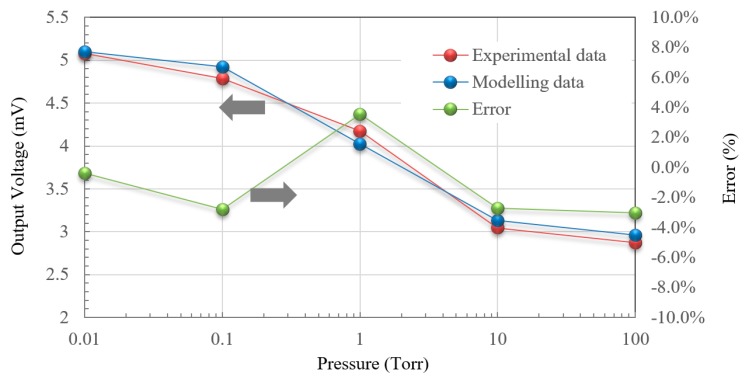
Experimental data versus analysis of modelling for modulation frequencies at 1 Hz.

Seen in Figure 7, the response curve (on a logarithmic scale) at 1 Hz also was analyzed via curve fitting with the modelling using Equation (13), which led to the transition pressure of 10 Torr. The parameter extraction gives an excellent description and agreement with measurement. The error is less than ±4%. It means our proposed measurement by frequency modulation with a proper offset of bias voltage applied heating could dramatically match and suit for the application and operation of a thermoelectric vacuum sensor.

### 4.3. Sensitivity of Thermoelectric Vacuum Sensor

**Figure 8:**
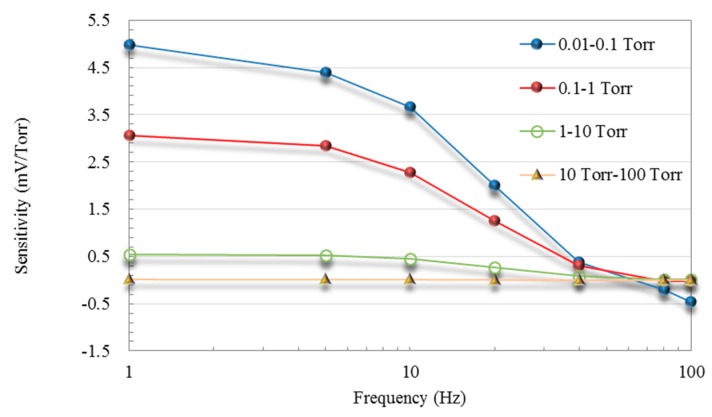
Sensitivity vs. frequency across various ranges of vacuum pressure.

To continue the investigation of vacuum measurement, the sensitivity of the proposed vacuum sensor, shown as the slope of curves across four vacuum pressure ranges in Figure 6, was analyzed. It is evaluated according to $S=\frac{\Delta V}{\Delta P}$, which reveals the sensitivity over a pressure range Δ*P*. The original output voltage of the thermopile was derived from the output signal and the gain of the amplification circuit. Indicated by the curves in Figure 8, the sensitivity of the vacuum sensor in the ranges of 0.01–0.1 Torr and 0.1–1 Torr are nearly six–ten times that in other ranges, which means that the proposed thermoelectric vacuum sensor provides superior sensitivity under medium-vacuum conditions. Note that the maximum sensitivity of the proposed sensor exceeded the sensitivity of the CMOS-compatible micro-Pirani vacuum sensor proposed by the other researchers [2].

Figure 5, Figure 6, Figure 7 and Figure 8 show that under medium-vacuum conditions (i.e., 25–10^−3^ Torr), the proposed sensor provided good sensitivity and resolution when modulating the frequency to less than 10 Hz.

**Figure 9:**
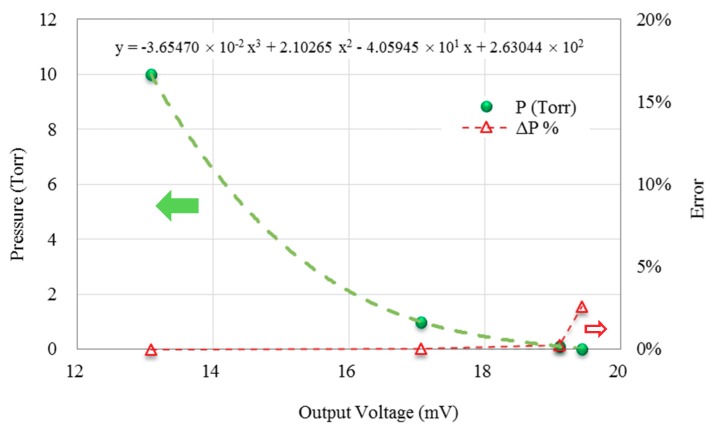
Calibration and error analysis of vacuum measurement at 10 Hz.

Moreover, it is important to evaluate the performance of the proposed vacuum measurement scheme via the error analysis. The calibration for vacuum measurement was kept with an appropriate heating frequency setup at 10 Hz. The measurement with low frequency near a DC condition was easy to affect by ambient temperature and background noises. Figure 9 presents calibration curves as a function of output voltage, as an indicator of error performance, with the heating frequency set at 10 Hz across a range of vacuum conditions. Between 0.1 and 10 Torr, the degree of error was consistently below 0.25%, which shows an excellent characteristic curve for measurement. Meanwhile the maximum degree of error was 2.59%, which happened at 0.01 Torr.

**Figure 10:**
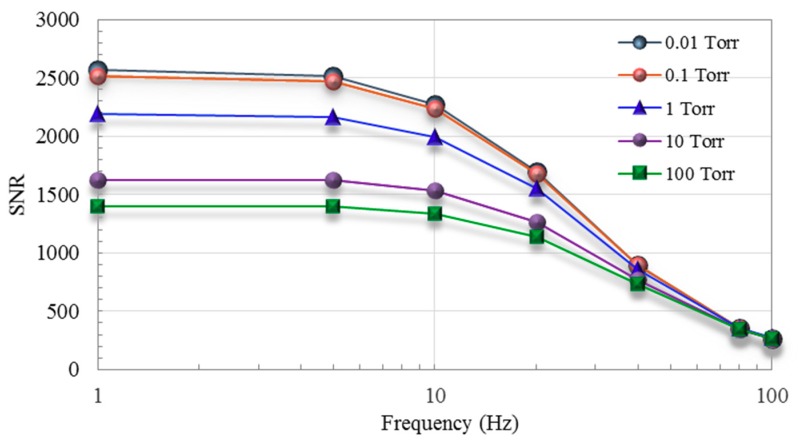
Signal-to-noise ratio (SNR) versus frequency for different pressures.

Figure 10 presents signal-to-noise ratio (SNR) curves in the range of 0.01–100 Torr. To calculate the SNR values, the original output signal and Johnson’s noise from the thermoelectric elements were adopted. The SNR values increased with a decrease in frequency, from >1138 (at 20 Hz, 100 Torr) to 2568 (at 1 Hz, 0.01 Torr). Seen at frequencies below 20 Hz, the sensitivity was also significantly higher. Based on asymmetrical applied heating (i.e., setting *V*_off_) to the active heater, these results indicate that the proposed sensor operates more effectively under a lower first harmonic modulation frequency. Table 2 summarizes the performance parameters of the proposed thermoelectric vacuum sensor.

### 4.4. Responsivity and Heating Power

**Figure 11:**
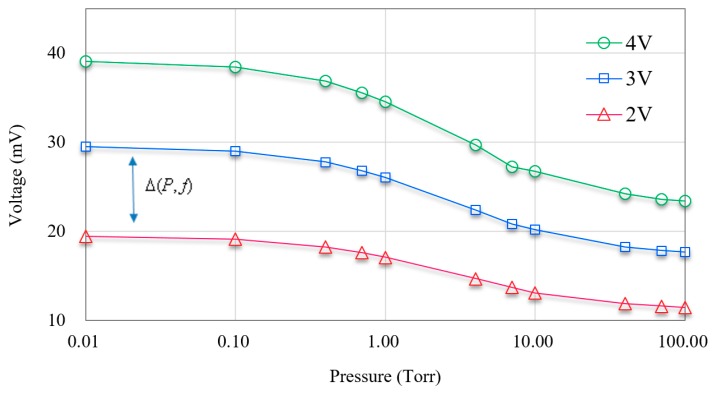
Output voltage versus pressure under various bias voltages applied to heater at 10 Hz.

We also sought to elucidate the relationship between heating power and the thermopile responsivity. Using a heating frequency of 10 Hz, the output voltage of the sensor was compared using a range of input bias voltages for the heater (2 V, 3 V and 4 V). Shown in Figure 11, the output voltage from the PLL increased with the bias voltage applied to the heater.

Note the equal spacing between the output voltage curves exists among the entire pressure range (0.01–100 Torr), indicating that these results correspond with the theoretical values derived using Equation (14).

## 5. Conclusions

This paper presents a CMOS–MEMS thermoelectric vacuum sensor based on a frequency modulation technique. The proposed thermopile was fabricated using 64 pairs of thermocouples based on the TSMC 0.35 μm CMOS process via post-MEMS processing. Based on an asymmetrical signal, the periodic bias voltage with first harmonic modulation frequency and offset voltage was applied to the heater. The frequency response of the sensor was assessed at frequencies of 1–100 Hz. The output voltage from the thermoelectric sensor (with the same locked frequency) was amplified by a signal processing circuit and then sent to a PLL for modulation. Following calibration, the error rate performance was excellent (<0.25%) across a pressure range of 0.1–10 Torr. Note also that the sensitivity of the system was far higher when using a heating frequency of less than 10 Hz. The sensitivity and the SNR values revealed that the proposed sensor operated more effectively under a lower heating frequency. Through the offset of applied bias voltage, our mathematical model of gas thermal conductance under frequency modulation was in good agreement with the experiment measurement. The important thermal parameter, transition pressure, of gas thermal conductance was extracted and verified by our frequency modulation technique. It gives a new approach for more investigations and applications of vacuum sensing.

## Figures and Tables

**Table 1:** Design parameters for proposed thermoelectric vacuum sensor.

Parameter	Typical Values
Chip size	1500 μm × 1500 μm
Diameter of membrane	680 μm
Number of thermocouples	64 pairs
Size of heater	460 μm × 5 μm
Size of etching window	200 μm × 4.5 μm
Size of thermocouples	250 μm × 20 μm

**Table 2:** Parameters of proposed thermoelectric vacuum sensor.

Parameter	Typical Values	Unit
Sensitive area	680 × 680	mm^2^
Sensitivity	4.98 ^1^	mV/Torr
Resistance	14.8	kΩ
SNR	2568 ^1^	-
Time constant	20	ms

^1^ Maximum value.
